# Is discontinuation of clopidogrel necessary for intracapsular hip fracture surgery? Analysis of 102 hemiarthroplasties

**DOI:** 10.1007/s10195-013-0235-1

**Published:** 2013-04-06

**Authors:** Fahad S. Hossain, Rohit Rambani, Helen Ribee, Lutz Koch

**Affiliations:** 1 North Lincolnshire and Goole Hospitals NHS Foundation Trust, Grimsby, UK; 2 The Mid Yorkshire Hospitals NHS Trust, Dewsbury, UK; 3 23 Kings Court, Hull, HU2 8JR UK; 4 Leeds Teaching Hospitals NHS Trust, Leeds, UK

**Keywords:** Clopidogrel, Hip fracture, Transfusion, Complications

## Abstract

**Background:**

An increasing number of elderly patients are managed with long-term antiplatelet therapy. Such patients often present with hip fracture requiring surgical intervention and may be at increased risk of perioperative bleeding and complications. The aim of this study was to ascertain whether it is necessary to stop clopidogrel preoperatively to avoid postoperative complications following hip hemiarthroplasty surgery in patients with intracapsular hip fracture.

**Materials and methods:**

A retrospective review of 102 patients with intracapsular hip fracture with either perioperative clopidogrel therapy [clopidogrel group (CG)] or no previous clopidogrel exposure [no clopidogrel group (NCG)] who underwent hip hemiarthroplasty surgery was undertaken. Statistical comparison on pre- and postoperative haemoglobin, American Society of Anesthesiologists (ASA) grade, comorbidities, operative time, transfusion requirements, hospital length of stay (LOS), wound infection, haematoma and reoperation rate between the two groups was undertaken. Regression analysis was undertaken to ascertain the risk ratios (RR) of complications and transfusion associated with clopidogrel.

**Results:**

There was no difference with respect to ASA grade, comorbidities (except cardiac comorbidities), pre- and postoperative haemoglobin levels, operation time, age or gender between the two groups. Four and two patients, respectively, required transfusion postoperatively in the CG and NCG (*p* = 0.37). There was no difference with respect to LOS, wound infection, haematoma or reoperation rate between the two groups postoperatively. The covariate-adjusted RR for complications and transfusion while being on clopidogrel were 0.43 [95 % confidence interval (CI) 0.07–2.60] and 3.96 (95 % CI 0.40–39.68), respectively.

**Conclusion:**

Continuing clopidogrel therapy throughout the perioperative period in patients with intracapsular hip fracture is not associated with an increased risk of complications following hip hemiarthroplasty surgery.

## Introduction

Mortality associated with fragility hip fractures is high, with more than a third of patients dying within 12 months [1]. Most of these deaths are due to medical causes related to concurrent comorbidities [2]. Over 65 % of hip fracture patients have an American Society of Anesthesiologists (ASA) score of 3 or above, further reflecting the high prevalence of coexisting comorbidities [3], of which ischaemic heart disease is common. In fact, previous cohort studies have suggested a genetic link between a diagnosis of cardiovascular disease and subsequent development of fragility hip fractures [4, 5]. It is therefore not surprising that a significant proportion of these elderly patients will be on antiplatelet therapy at presentation with a hip fracture.

Clopidogrel irreversibly inhibits platelet aggregation which continues throughout the lifespan of the platelets affected [6]. It is superior to aspirin in secondary prevention of ischaemic stroke, myocardial infarction or vascular death [7], with dual therapy using clopidogrel and aspirin being most effective in the management of vascular events particularly in patients following coronary stenting [8, 9]. While the therapeutic advantages of clopidogrel therapy are evident, its continuation during the perioperative phase has its disadvantages. With regards to cardiac surgery specifically, continued clopidogrel therapy has resulted in increased perioperative blood loss, haemorrhagic complications, transfusion requirement and infection [10, 11]. The potential risk of haematoma associated with regional anaesthesia techniques [12] in orthopaedic patients, reinforces the rationale for disruption in antiplatelet therapy with ensuing delay in surgery. Concerns with hip fracture patients on concomitant clopidogrel therapy have often led to its discontinuation with subsequent delay prior to definitive surgical treatment. Previous studies have shown that up to 21 % of orthopaedic surgeons and more than 40 % of orthopaedic departments in the UK follow a policy of preoperative interruption in clopidogrel therapy for 5–10 days in hip fracture patients [13, 14].

In contrast, however, discontinuation of clopidogrel therapy perioperatively may result in considerable therapeutic and surgery-related disadvantages. Acute withdrawal of clopidogrel results in a prothrombotic and proinflammatory state that may complicate surgery, resulting in adverse clinical events [15]. Furthermore, cessation of clopidogrel therapy for at least 5 days is required to allow return of platelet function adequate for implementation of regional anaesthesia in hip fracture surgery [16]. This in itself is of major concern, as surgical delay in hip fracture management of more than 2 days is associated with a significantly increased risk of complications and mortality within 30 days and 1 year [17, 18]. From a cardiac perspective, discontinuation of clopidogrel is associated with an increased incidence of myocardial infarction or mortality in patients treated either medically or with coronary stenting for acute coronary syndrome. In both groups over half the events occurred within 90 days of stopping clopidogrel therapy [19].

There is a paucity of information on the risks of continuing clopidogrel perioperatively in hip fracture surgery with a lack of consensus on this issue amongst surgeons [13]. We therefore aimed to ascertain whether there were any differences in blood transfusion, wound complications and reoperations during the postoperative period between patients who continued their long-term clopidogrel therapy throughout the perioperative period and those who were not on clopidogrel.

## Materials and methods

Patients were retrospectively identified from an audit database of hip fractures at our institution between April 2008 and April 2010 with verification of clopidogrel exposure from electronic discharge summaries detailing medication history. Informed consent was obtained prior to patient inclusion in our study. The study was planned and implemented in accordance with the ethical standards of the Helsinki Declaration as amended in 2000 and was approved by our institution’s clinical governance committee. We included all patients over the age of 50 years with a displaced intracapsular fracture treated surgically within 48 h of admission. We excluded patients with extracapsular fractures, polytrauma, pathological fractures, coagulopathies, haematological malignancies, pre-existing warfarin or dipyridamole therapy, thrombocytopaenia on admission (platelet count <150 × 10^9^/L), recent active bleeding or gastrointestinal ulcers.

Case notes were reviewed to collect data on age, gender, ASA grade, presence of individual comorbidities and preoperative haemoglobin (Hb) levels in all patients. We also collected surgery-related data including operation duration and use of cement. Our outcome measures included postoperative Hb levels, transfusion rates, incidence of wound infection, symptomatic haematomas, reoperations for any cause and hospital length of stay (LOS). We reviewed patient anaesthetic charts to determine the type of anaesthesia that was given (i.e. general or regional anaesthesia) and furthermore if there were any ensuing anaesthetic complications.

We identified consecutive patients who were surgically treated while long-term clopidogrel therapy was continued [intervention group (CG)] and consecutive patients without any history of clopidogrel exposure [control group (NCG)]. Patients in both groups may or may not have been on additional aspirin therapy.

Preoperative medical optimisation prior to surgery was undertaken via multidisciplinary consultations. Patients in both groups were treated with hip hemiarthroplasty using a lateral approach. Our cemented prostheses of choice were either the Furlong^®^ cemented hemiarthroplasty (JRI, Sheffield, UK) or the Thompson hemiarthroplasty (Depuy, Leeds, UK). Our uncemented prosthesis of choice was the hydroxyapatite-coated Furlong^®^ H-A.C hemiarthroplasty (JRI, Sheffield, UK). The Austin–Moore hemiarthroplasty (Depuy, Leeds, UK) was reserved for older patients with poor pre-injury mobility and higher perioperative risks. Antibiotic prophylaxis was undertaken with 1 g of intravenous flucloxacillin (or vancomycin in cases of penicillin allergy) at anaesthetic induction and two further postoperative doses. All patients received thromboprophylaxis with 40 mg of enoxaparin postoperatively for 6 weeks administered subcutaneously. A multidisciplinary approach to postoperative care with input from orthogeriatricians, physiotherapists and occupational therapists were undertaken prior to discharge of patients to their own home or a community rehabilitation facility. They were encouraged to ambulate immediately as pain allowed postoperatively. All patients were subjected to a repeat pelvis radiograph and blood tests at 48 h postoperatively.

All categorical variables were analysed using the chi-squared or Fisher’s exact test, while the independent-samples Students *t*/Mann–Whitney test was used for continuous variables when comparing the two groups in univariate analysis. We combined all complications of haematoma, wound infection or reoperation for any reason into a binary variable (1 = any complication occurred, 0 = no complication occurred). In so doing we did not account for the severity of the complications. Any other approach which would have accounted for severity of complications would make continuing clopidogrel appear more protective against complications, hence introducing bias in favour of its continuation. The need for transfusion was also measured as a binary variable (1 = transfusion needed, 0 = transfusion not needed). We compared the outcomes of these binary variables of complication and transfusion using a working Poisson regression model adjusting for covariates. We chose parameters which had a significance level of *p* < 0.15 on initial univariate analysis as covariates [20]. The use of the working Poisson model allows direct estimation of relative risk, which is easier to interpret and more relevant clinically [21, 22]. It is hence preferred over logistic regression, which estimates odds ratios and has been used for relative risk estimation in previous cohort studies [23].

## Results

There were 50 and 52 patients in the CG and NCG, respectively. The demographic and comorbidity-related data are summarised in Table 1, while data pertaining to surgery-related factors and outcome variables are summarised in Tables 2 and 3, respectively. The mean ages in the CG and NCG were 82.8 ± 7.3 years and 83.2 ± 7.8 years, respectively (*p* = 0.82). There was no statistically significant difference with respect to gender (*p* = 0.87) or ASA grade (*p* = 0.085) between the two groups. The mean preoperative Hb levels were 12.5 ± 1.4 and 12.6 ± 1.2 g/dL, respectively, in the CG and NCG (*p* = 0.72). There was no statistically significant difference in comorbidities between the two groups except that of cardiovascular disease (*p* < 0.0001) which was higher in the CG compared with the NCG. Similarly there was no statistically significant difference in the proportion of patients on concomitant aspirin between the two groups (*p* = 0.14) (Table 1).

**Table 1:** Patient characteristic and comorbidity data for both groups

Patient characteristic	CG	NCG	*p*-Value
Age (mean ± SD)	82.8 ± 7.3 years	83.2 ± 7.8 years	0.82
Gender (male:female)	1:4.6	1:4.2	0.87
ASA grade, number (%)			
ASA 1	–	4 (7.7)	0.085*
ASA 2	16 (32)	11 (21.2)	
ASA 3	29 (58)	27 (51.9)	
ASA 4	5 (10)	10 (19.2)	
ASA 5	–	–	
Preoperative Hb (mean ± SD)	12.5 ± 1.4 g/dL	12.6 ± 1.2 g/dL	0.72
Comorbidities, number (%)			
Diabetes mellitus	5 (10)	5 (9.6)	0.60
Hypertension	21 (42)	22 (42.3)	0.57
Respiratory disease	3 (6)	6 (11.5)	0.26
Renal disease	5 (10)	8 (15.4)	0.30
Thyroid disease	2 (4)	4 (7.7)	0.36
Anaemia	15 (30)	14 (26.9)	0.45
Cardiovascular disease	46 (92)	8 (15.4)	<0.0001*
Cancer	5 (10)	3 (5.8)	0.48
Thromboembolic disease	5 (10)	4 (7.7)	0.48
Cerebrovascular disease	8 (16)	6 (11.5)	0.36
Dementia	6 (12)	12 (23.1)	0.11*
Aspirin intake	16 (32)	23 (44.2)	0.14*

*SD* standard deviation

* Included in regression analysis

**Table 2:** Surgical and hospital episode-related factors for both groups

Surgical or hospital parameter	CG	NCG	*p*-Value
Cement usage, number (%)	22 (44)	10 (19.2)	0.01*
Length of surgery (mean ± SD)	50.5 ± 11.4 min	55.5 ± 15.9 min	0.072*
LOS (mean ± SD)	17.3 ± 13.3 days	20.5 ± 16.6 days	0.28
Anaesthesia type [number (%)]			
General	44 (88)	18 (34.6)	
Regional	6 (12)	34 (65.4)	

*SD* standard deviation

* Included in regression analysis

**Table 3:** Outcome parameters for both groups

Outcome parameter	CG	NCG	*p*-Value
Postoperative Hb, mean ± SD	10.8 ± 1.5 g/dL	11.1 ± 1.5 g/dL	0.37
Transfusion given, number (%)	4 (8)	2 (3.85)	0.37
Total no. of units	8	5	
Mean no. of units	2 ± 0	2.5 ± 0.71	0.16
Time to transfusion	3 ± 1.8 days	3.5 ± 0.7 days	
Adjusted risk ratio (95 % CI)	3.96 (0.40–39.68)		
Complication—yes/no, number (%)	4	4	1.0
Haematoma	3	1	0.36
Wound infection	1	2	1.0
Reoperation	1	2	1.0
Adjusted risk ratio (95 % CI)	0.43 (0.07–2.60)		

*SD* standard deviation

There were 7 and 15 patients who had an Austin–Moore prosthesis implanted in the CG and NCG, respectively. There was a higher proportion of cemented procedures in the CG compared with the NCG (*p* = 0.01). However, there was no statistically significant difference with respect to operation duration between the two groups (*p* = 0.072). Similarly there was no statistically significant difference in mean hospital LOS between the two groups (*p* = 0.28) (Table 2).

The mean postoperative Hb levels were 10.8 ± 1.5 and 11.1 ± 1.5 g/dL in the CG and NCG (*p* = 0.37). Four patients in the CG required a transfusion as compared with two patients in the NCG. A total of 8 blood units were utilised in the CG while 5 in the NCG. The mean number of units used was 2 ± 0 and 2.5 ± 0.71 in the CG and NCG, respectively (*p* = 0.16). The covariate-adjusted relative risk (RR) of having a blood transfusion while continuing clopidogrel was 3.96 (95 % CI 0.40–39.68).

There were three cases of symptomatic haematomas in the CG with one in the NCG (*p* = 0.36). The overall wound infection rate for both groups was 2.94 %, with one and two patients developing wound infections in the CG and NCG, respectively (*p* = 1.0). One patient in the CG had a reoperation for evacuation of a haematoma. In the NCG, one patient had a reoperation for a deep wound infection, while another returned to the operating room for reduction of a dislocated hip. The RR for all complications combined while continuing clopidogrel after adjusting for covariates was 0.43 (95 % CI 0.07–2.60) (Table 3).

General anaesthesia was more common in the CG with 88 % of patients receiving a general anaesthetic, while 65.4 % of patients in the NCG received regional anaesthesia (Table 2). There were no anaesthetic-related complications in either group.

## Discussion

Fragility hip fractures are common in patients with multiple comorbidities including that of cardiovascular disease. Our cohort study demonstrates that continued clopidogrel antiplatelet therapy during the perioperative period in such patients does not result in worse outcomes as compared with a perioperative period in intracapsular hip fracture patients who have never been exposed to clopidogrel. The overall transfusion and complication rates were low with no statistically significant difference between the two groups.

Coronary dilatation for cardiovascular disease is common, accounting for over 2 million cases annually in Western countries [24], of which the majority are percutaneous stenting procedures with subsequent long-term clopidogrel therapy. Approximately 5 % of these patients are likely to be subjected to non-cardiac surgery within a year following such interventions [25]. In fact, the indications for clopidogrel therapy extend beyond that of just patients following coronary stenting to that of other cardiac and vascular conditions. As such the proportion of patients on long-term clopidogrel therapy who are subjected to non-cardiac surgery including orthopaedic procedures may be even higher. In our study both the CG and NCG were well matched with respect to age, gender, ASA grade and preoperative haemoglobin levels, confirming similarity of the patient profile between the two groups. The mean age was over 80 years with high prevalence of ASA grade 3 in both groups. These findings are similar to that of the wider National Hip Fracture Database (NHFD), which incorporates hip fracture patients from the majority of healthcare institutions within the UK [3]. There were no differences with respect to the prevalence of any of the individual comorbidities except that of cardiovascular disease. This was expectedly higher, with over 90 % of patients in the CG suffering with it.

While it is assumed that prolonged surgery is associated with increased intra-operative bleeding, it is also entirely possible that a tendency for increased intra-operative bleeding may prolong surgical duration owing to extra attention and time needed for achieving adequate haemostasis and an optimum operative field. Surgical times were similar with mean operative duration times of less than an hour in both groups. We hence speculate that intraoperative bleeding in the CG was perhaps only marginally higher or at best similar to that of the NCG and hence unlikely to account for prolonged surgery. We do, however, acknowledge that the determinants of surgical duration are multifactorial including surgical experience and other patient factors such as habitus and body mass index (BMI). Use of a cemented prosthesis was more common in the CG as compared with the NCG, which is further explained by the higher proportion of Austin–Moore prostheses used in the NCG. This interestingly implies that a higher proportion of patients in the NCG were considered to have increased perioperative risk with poor pre-injury mobility. Cemented hemiarthroplasties have equivalent outcomes with similar complication rates compared with hydroxyapatite-coated uncemented hemiarthroplasties [26–28], and their use in our study simply reflects supervising surgeon preference.

The mean postoperative haemoglobin levels were similar in the two groups. The drop in mean haemoglobin levels from surgery was 1.66 and 1.49 g/dL in the CG and NCG, respectively. The changes are modest compared with that of others evaluating blood loss from hip fracture surgery while on antiplatelet therapy of aspirin or clopidogrel [29, 30]. These studies, however, attribute extracapsular fracture patterns as a cause for such increased blood loss. The exclusion of extracapsular fractures from our study cohorts may explain the comparatively smaller changes in haemoglobin levels postoperatively. There was no difference in transfusion rates between the two groups. The relative risk estimate of transfusion while on clopidogrel after adjustment for covariates was not significant at 3.96 (95 % CI 0.40–39.68). Our results are in agreement with that of the study by Nydick et al. [31], which showed no difference in transfusion rates between patients continued on clopidogrel and those who were not during acute trauma orthopaedic surgery of both upper and lower limbs. Similarly, a presentation given at the annual meeting of the Orthopaedic Trauma Association in 2008 found no difference in bleeding complications or transfusion requirement in a cohort of 498 patients with either clopidogrel, aspirin or no antiplatelet therapy during hip fracture treatment [32]. In contrast, previous studies have shown increased bleeding and transfusion complications while continuing clopidogrel during cardiac surgery [11]. These findings should, however, be interpreted in light of the fact that cardiac surgery is normally conducted with full intraoperative heparinisation for cardiopulmonary bypass, which will contribute to such complications.

Although not statistically significant, the trends were in favour of higher symptomatic haematomas while on perioperative clopidogrel in our study. Chechik et al. [33] in their matched cohort study demonstrated an increase in hidden blood loss in patients on clopidogrel or dual antiplatelet therapy during hip fracture surgery but failed to report any significant difference in transfusion rates for the same. Our findings are consistent with this where the higher incidence of symptomatic haematomas associated with perioperative clopidogrel continuation may be analogous to hidden blood loss into third spaces postoperatively.

Infection rates were low in our study with overall incidence of 2.94 %, with one patient from the NCG developing a deep wound infection necessitating surgical washout. This accounted for <1 % of the whole study population. Our results are comparable to those of previous studies demonstrating deep wound infection rates of 1.2–1.8 % in hip fracture surgery [34–36]. There was no difference in infection or reoperation rates between the two groups. In fact, one patient had a reoperation for dislocation, which may be considered to be unrelated to antiplatelet therapy. The covariate-adjusted relative risk for overall complication while continuing clopidogrel was not significant at 0.43 (95 % CI 0.07–2.60) but may suggest a trend towards a protective effect. Our findings are in conformity with those of Nydick et al. and Chechik et al. [31, 33], who showed no difference in wound infection rates between patients continued on clopidogrel perioperatively and those who were not during trauma orthopaedic surgery. In contrast, continuation of clopidogrel as part of dual antiplatelet therapy during cardiac surgery has been associated with increased infection rates [10]. As previously mentioned, cardiac surgery is confounded by intraoperative heparinisation, which may contribute to subsequent complications including infection. Furthermore, reports of increased infection rates associated with the use of low-molecular-weight heparin for thromboprophylaxis after hip fracture surgery further supports this notion [37].

There was a significantly larger proportion of patients who underwent general anaesthesia in the CG compared with the NCG. This may be attributed to the perceived risks of regional anaesthesia in patients on antiplatelet therapy. Only six patients underwent regional anaesthesia in the CG. None of these patients were on dual antiplatelet therapy. Our study is in keeping with the recommended guidelines regarding implementation of regional anaesthesia in patients on antiplatelet therapy [12]. There were no anaesthetic-related complications in either group. Use of regional anaesthesia in hip fracture patients is often preferred due to the presence of multiple comorbidities and limited physiological reserve to withstand general anaesthesia. However, the absence of any complications in the use of general anaesthesia in the CG supports the relative safety of its use. A previous meta-analysis suggested some advantage in the use of regional anaesthesia in terms of reducing postoperative confusion but failed to conclude on any difference with respect to mortality or other outcomes between the two anaesthetic types [38]. A recent randomised prospective study comparing general and regional anaesthesia in hip fracture patients showed similar findings of no difference in mortality but increased postoperative complications and pain with the use of general anaesthesia [39]. The advantages of early surgery within 48 h are well established for hip fracture management in terms of reducing the risk of death [17, 18, 40], while in contrast the use of regional anaesthesia in hip fracture surgery does not confer any added benefit in terms of reducing mortality. Hence, the benefits of its use in hip fracture patients on concomitant clopidogrel therapy where a delay of >48 h is required prior to definitive surgical management is outweighed by the benefits of reduced mortality risk for expedited surgery under general anaesthesia.

Our study is limited by its retrospective design and relatively small sample size. We did, however, identify our patient samples from a wider institutional audit database of hip fracture patients with strict exclusion criteria in order to maintain a homogeneous sample. As a result we excluded extracapsular fractures, and perhaps their inclusion in a separate analysis would have allowed us to draw conclusions applicable to the wider population of patients with different hip fracture patterns.

In conclusion, our data supports the argument for continuing clopidogrel throughout the perioperative period of hip hemiarthroplasty surgery in high-risk patients where the risks of its discontinuation may be associated with significant morbidity and even an increased risk of mortality [41]. We observed no increased risk of complications in the cohort continued on their long-term clopidogrel therapy perioperatively. Use of general anaesthesia in such patients can be safely implemented, avoiding the potential problems of regional anaesthesia related to antiplatelet therapy. However, we recognise that complex pathophysiological interactions may be involved in the anaesthetic and perioperative management of such elderly and comorbid patients, and as such an individualised approach in dealing with perioperative clopidogrel therapy is required. Nonetheless, larger prospective comparative studies assessing the outcomes of hip fracture surgery while on concomitant clopidogrel therapy are still required to strengthen the evidence for such clinical practice.
